# Development of global monthly dataset of CMIP6 climate variables for estimating evapotranspiration

**DOI:** 10.1038/s41597-023-02475-7

**Published:** 2023-08-26

**Authors:** Young Hoon Song, Eun-Sung Chung, Shamsuddin Shahid, Yeonjoo Kim, Dongkyun Kim

**Affiliations:** 1 Department of Civil Engineering, Seoul National University of Science and Technology, Nowon-gu, 01811 Seoul, South Korea; 2 School of Civil Engineering, Universiti Teknologi Malaysia (UTM), 81310 Skudai, Johor Malaysia; 3 Department of Civil and Environmental Engineering, Yonsei University, 03722 Seoul, South Korea; 4 Department of Civil Engineering, Hongik University, 04066 Seoul, South Korea

**Keywords:** Hydrology, Climate change

## Abstract

Reliable projection of evapotranspiration (ET) is important for planning sustainable water management for the agriculture field in the context of climate change. A global dataset of monthly climate variables was generated to estimate potential ET (PET) using 14 General Circulation Models (GCMs) for four main shared socioeconomic pathways (SSPs). The generated dataset has a spatial resolution of 0.5° × 0.5° and a period ranging from 1950 to 2100 and can estimate historical and future PET using the Penman-Monteith method. Furthermore, this dataset can be applied to various PET estimation methods based on climate variables. This paper presents that the dataset generated to estimate future PET could reflect the greenhouse gas concentration level of the SSP scenarios in latitude bands. Therefore, this dataset can provide vital information for users to select appropriate GCMs for estimating reasonable PETs and help determine bias correction methods to reduce between observation and model based on the scale of climate variables in each GCM.

## Background & Summary

Evapotranspiration (ET) is an important component of the water cycle and plays a major role in agriculture and water management^1^. Earth systems have shown robust change signals of climate variables since the 20th century^2^. Recently, the climate crisis facing humanity has been mainly due to the changes in the water cycle caused by changing patterns of precipitation, temperature, and surface runoff^3^. Furthermore, the changes in the hydrological cycle due to an increase in the atmospheric water vapor content are related to extreme changes in the various factors involved in the general circulation^4^. Numerous studies have been conducted to characterize regional and continental scale surface water losses to the atmosphere by estimating potential ET (PET). The Food and Agriculture Organization (FAO) of the United Nations has recommended using the Penman-Monteith (PM) method to estimate potential evapotranspiration^5^, and some studies are used as reference models for other methods with less input data^6–9^. However, PM requires many climate variables to estimate PET. Therefore, climate models that simulate sufficient climate variables can only be used to estimate PET.

General circulation models (GCMs) have been widely used to estimate PET because many models simulate several climate variables for historical and future periods^10–17^. GCMs are continuously being updated, with the incorporation of new physical processes and biochemical cycles and simulations at higher spatial resolutions. However, CMIP6 GCMs have issues with Equilibrium Climate Sensitivity (ECS)^18^. Currently, some studies have provided scientific evidence for the reason for the high ECS of CMIP6, claiming that CMIP6 GCMs were more sensitive to greenhouse gases and exhibited stronger temperature increases than previous models during the 21st century due to enhanced cloud feedback^19^. Many studies compared the performance of past reproducibility of CMIP6 GCMs with their previous versions in terms of various evaluation metrics and showed their better performance than the earlier versions^20–26^. The advance in these model performances has made strides in providing scenarios for better future climate projection. Furthermore, Shared Socioeconomic Pathways (SSPs) representing the future greenhouse gas concentration of CMIP6 include future mitigation, adaptation, and efforts on climate change future social and economic changes based on the radiative forcing levels of Representative Concentration Pathways (RCPs)^27^. Therefore, SSP scenarios are an excellent source for exploring climate change by estimating realistic climates.

Climate impact assessment using newly presented SSPs is documented in IPCC’s assessment report 6. These concepts were provided in ScenarioMIP, an improved version of the integrated assessment models (IAM) based on change by anthropogenic causes, such as land use, greenhouse gas, and aerosol^27,28^. Furthermore, these concepts of scenarios would also reduce the knowledge gap of radiative forcing and temperature overshoots in the future.

**Fig. 1:**
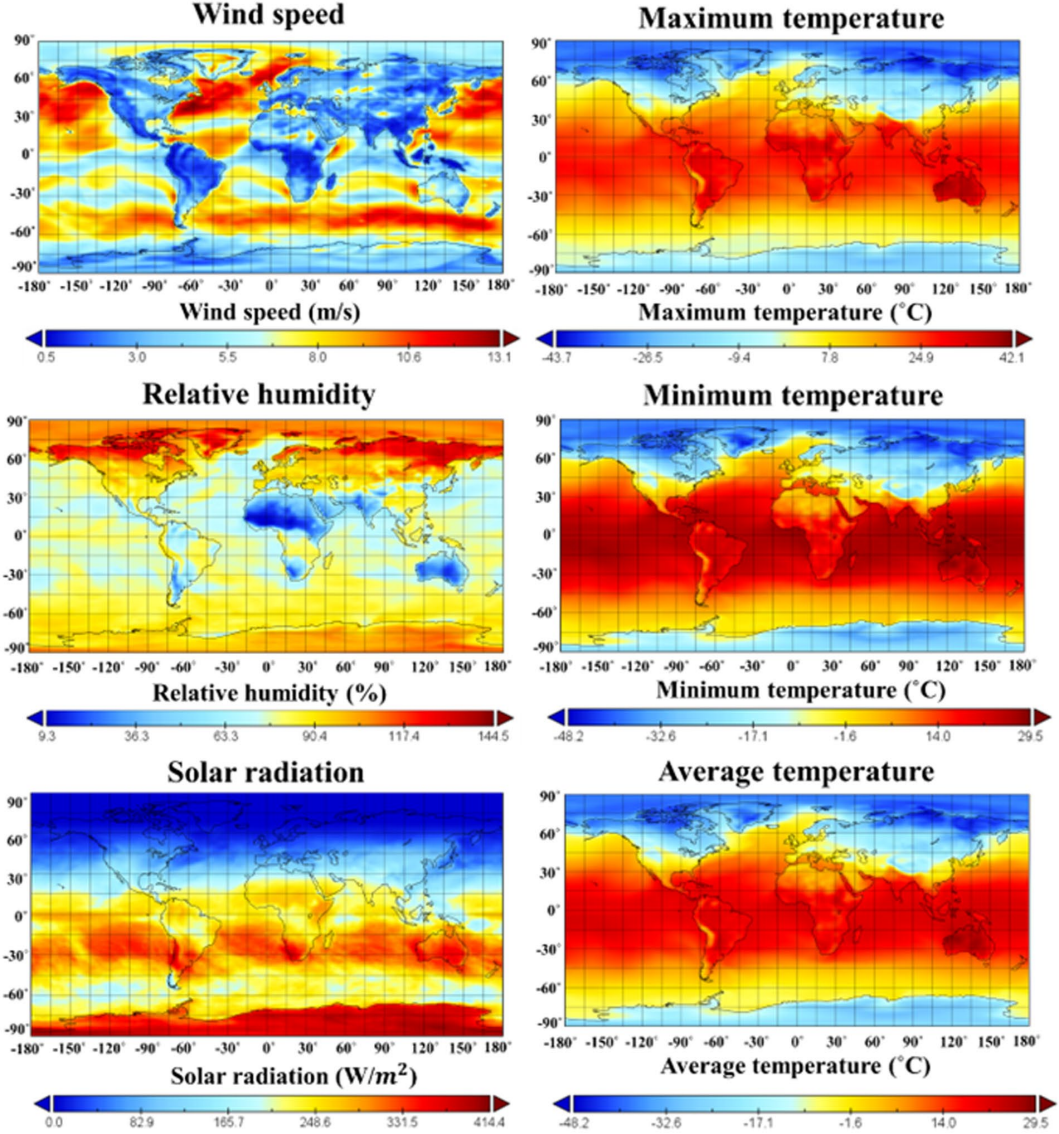
Example of ACCESS-CM2 dataset over the global scale (0°E-360°E in latitude and 0°N-90°N in longitude) in the historical period (1950–2014).

This study generates a raw types dataset of monthly climate variables (average temperature, minimum temperature, maximum temperature, wind speed, relative humidity, solar radiation) to estimate the global PET of 14 CMIP6 GCMs for the four main SSPs (Fig. 1 presents six climate variables represented by ACCESS-CM2 model as an example). Global climate data were re-gridded to 0.5° spatial resolution using linear interpolation. The re-gridded dataset of the CMIP6 GCM is freely available online in NetCDF format^29^. The generated climate variables can also be used to estimate global PET using Python code available in the ‘pyeto’ package^30^. The PET code is also provided in a ‘py’ file format^29^. The Penman-Monteith (PM) used in this study requires location (latitude and longitude), temperature, humidity, radiation, and windspeed for estimating evapotranspiration. Especially this dataset is needed to adjust some weather parameters for the local average value of atmospheric pressure, and it was used to compute extraterrestrial radiation and daylight hours. Latitude is directly involved in the calculation process of extraterrestrial radiation and daylight hours. The detailed description of the computational process in Penman-Monteith (PM) can be found under ‘pyeto’ in the Python archive. The projection period was divided into the near (2031–2065) and far (2066–2100) futures, and PET changes for both futures compared to the base period (1985–2014) were calculated. The projected future evapotranspiration using Python code and dataset increased in most scenarios. These results mean that the thermal energy for the future water cycle increases as the greenhouse gas concentration increases.

## Methods

### Data

The dataset generated in this study provides six climate variables (Relative humidity: Hurs; Maximum temperature: tasmax; Minimum temperature: tasmin; Average temperature: tas; Solar radiation: rsds; Windspeed: sfcWind) of 14 CMIP6 GCM to estimate PET over the globe, as shown in Table 1. The raw data for CMIP6 GCMs used in this study were collected from the CMIP6 archive^31^. The future climate variables for four SSPs (SSP1-2.6, SSP2-4.5, SSP3-7.0, and SSP5-8.5) were used. GCM simulation for the historical and future periods was re-gridded to 0.5° × 0.5° resolution using linear interpolation. The users can confirm a metadata summary in xlsx file format^27^. The variant label of the dataset was the r1i1pif1.

**Table 1:** Information on the GCMs used in this study.

Models	Resolution	Variant label	Climate variables
ACCESS-CM2	0.5° × 0.5°	r1i1p1f1	Relativue humidity (Hurs); Maximum temperature (tasmax); Minimum temperature (tasmin); Average temperature (tas); Solar radiation (rsds); Windspeed (sfcWind)
ACCESS-ESM1-5			
CanESM5			
CAS-ESM2-0			
CMCC-ESM2			
FGOALS-g3			
GFDL-ESM4			
INM-CM4-8			
INM-CM5-0			
IPSL-CM6A-LR			
MIROC6			
MPI-ESM1-2-HR			
MPI-ESM1-2-LR			
MRI-ESM2-0			

### Potential evapotranspiration estimation method

The projected historical and future ET can be estimated using a Python code developed by us, and we developed it based on the Penman-Monteith (PM) method. PM method proposed by Allen *et al.*^5^, to estimate monthly global PET using six climate variables of CMIP6 GCMs. PM represents a standard value of PET. It can be calculated using Eq. (1), as below:

1 $$PET=\left(\frac{0.408\times \Delta \times \left({R}_{n}-G\right)+\gamma \left(\frac{900}{{T}_{av}+273}\right)\times {u}_{2}\left({e}_{s}-{e}_{a}\right)}{\Delta +\gamma \left(1+0.34{u}_{2}\right)}\right)$$

where *PET* is the monthly PM, *R*_*n*_ is the net radiation at the crop surface, *T*_*av*_ is the monthly mean temperature at a 2 m height (°C), *u*_2_ is the average monthly wind speed at a 2-m height (m/s), *e*_*s*_ is the saturation vapor pressure (kPa), *e*_*a*_ is the actual vapor pressure (kPa), Δ is the slope of the saturation vapor pressure versus temperature curve, *G* presents the soil heat flux density (MJ/*m*^2^*month*^−1^), and *γ* is the psychrometric constant.

## Data Records

The six climate variables of the CMIP6 GCM dataset for estimating potential evapotranspiration are available in NC-formatted files and can be freely downloaded from the repository^29^. Furthermore, a metadata summary of the available CMIP6 GCM dataset is provided as an xlxs formatted file from the repository^29^. Table 2 presents the information depending on each latitude in CMIP6 GCMs. The resolution of climate variables in the data archive was re-gridded as 0.5°× 0.5°. The global latitudes were separated into the six (e.g. L1: 0° to 29.5° and 0° to −29.5°, L2: 30° to 59.5° and 30° to −59.5°, L3: 60° to 90°). The six climate variables for the historical period span from 1950 to 2014. The projected climate variables were divided into the near (2031–2065) and the far (2066–2100) futures. The projected PM ETP for historical and future periods was estimated using six climate variables.

**Table 2:** Information of each latitude constructed in CMIP6 GCMs dataset.

Hemispheres	Latitude band	Range	Number of total grids
Northern (NH)	NL3	60° to 90°	14,641
	NL2	30° to 59.5°	22,004
	NL1	0° to 29.5°	14,918
Southern (SH)	SL1	0° to −29.5°	10,081
	SL2	−30° to −59.5°	2,118

## Technical Validation

The PM ETP dataset generated in this study was validated for similarity with Earth’s climate variables in latitude bands depending on the radiative forcing levels of SSPs. First, this study confirms the similarity in climate variables between 14 CMIP6 GCMs and Earth using statistical metrics. It validates the estimated historical evapotranspiration using the PM method based on the climate variables. Second, the projected climate variables for the future period were compared to confirm the projection model’s performance technology, and we verified that the projected future climate variables adequately reflected the greenhouse gas concentration levels of the SSP scenarios. Finally, we estimate the future evapotranspiration using PM based on the climate variables of SSPs, and the changes in PM ETP were calculated for the near and far futures compared to the historical period to confirm the relative changes based on the greenhouse gas concentration of SSPs.

### Validation of global climate variables and PM in the historical period

**Fig. 2:**
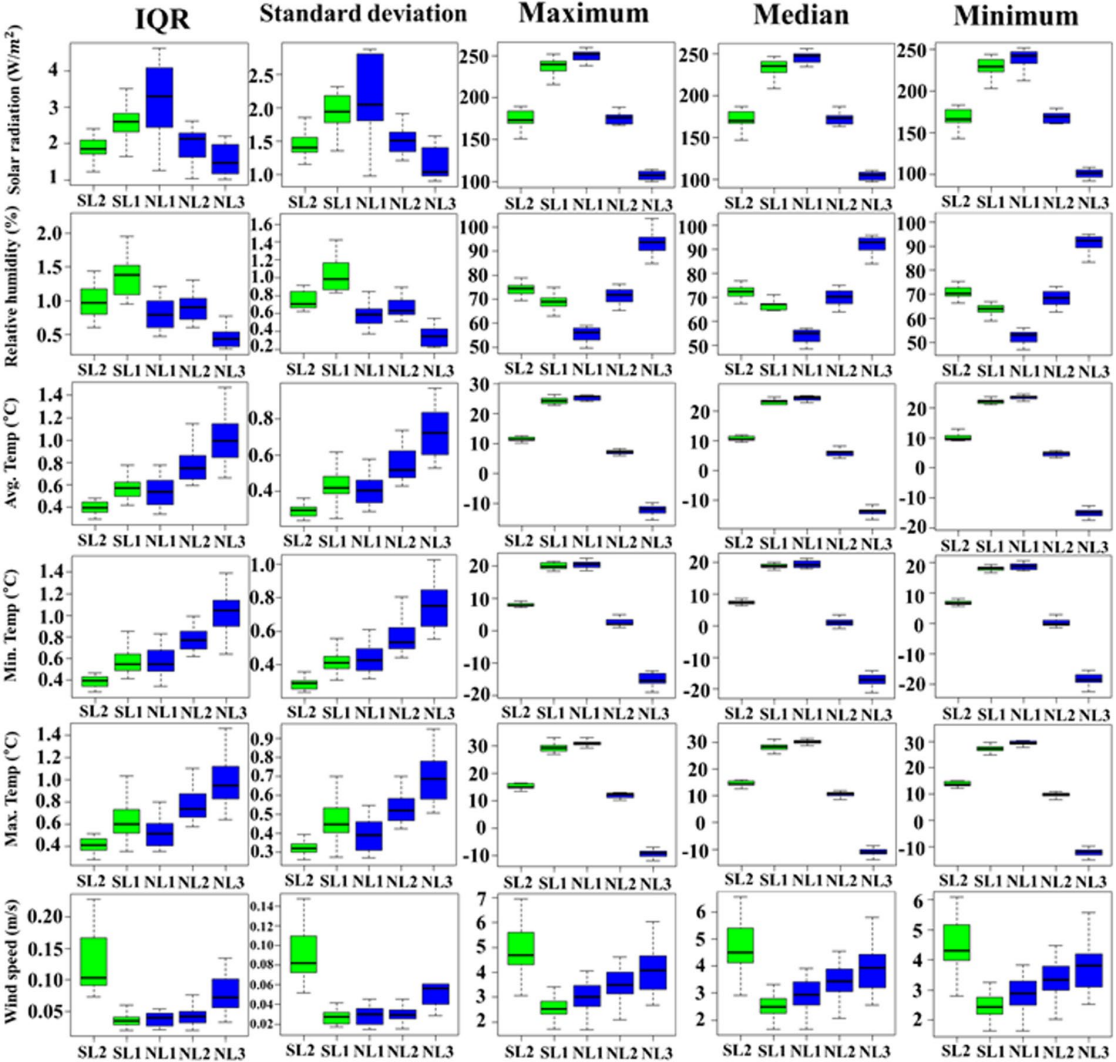
Statistical performance of six climate variables of 14 CMIP6 GCMs for the historical period (1950–2014) based on five metrics.

This study used five statistical metrics (Maximum, Minimum, Median, Standard deviation, and Interquartile range) to compare the range of climate variables in GCMs depending on the latitude bands. Figure 2 presents the statistical performance of the climate variables of 14 CMIP6 GCMs in simulating the historical climate for the five ranges of latitudes. The relative humidity in SL1 and NL1 (low latitudes in both hemispheres) was low compared to the other latitudes, whereas the relative humidity in NL3 was the highest. The maximum, minimum, and median relative humidity at NL1 was the lowest compared to the other latitudes, whereas the relative humidity in NL2 and NL3 showed the opposite results. The standard deviation and Interquartile range (IQR) were the highest at SL1, whereas those were lower at NL3 compared to the other latitudes. On the other hand, the maximum, minimum, and median wind speed was the lowest at SL1, whereas the wind speed in SL2 was higher than in other latitudes. The variability of wind speed was the highest in SL2. The solar radiation’s maximum, minimum, and median were the highest at low latitudes (SL1 and NL1) of both hemispheres and  the values in NL3 were the lowest than in other latitudes. Furthermore, solar radiation’s standard deviation and IQR were larger at low latitudes than at mid-latitudes of both hemispheres. The maximum, minimum, and average temperatures for the 14 CMIP6 GCMs were similar, with no significant differences between the GCMs. These results showed that the temperatures in low latitudes (SL1 and NL1) of both hemispheres were the highest, whereas the NL3 was the lowest. The standard deviation and IQR of temperatures at NH were also the largest compared to the other latitudes.

Table 3 shows the range of climate variables for the 14 CMIP6 GCMs in the historical period. Overall, the variability of  historical maximum temperature was the largest for FGOALS-g3, while IPSL-CM6A-LR had the lowest variability. In contrast, the variability of minimum temperature was the smallest in MRI-ESM2-0, while FGOALS-g3 had the highest variability. For average temperature, ACCESS-CM2 had the highest variability, while MRI-ESM2-0 had the lowest variability. Significantly, the relative humidity of CAS-ESM2-0 was anomalously larger than the other GCMs with an upper bound of 2628.1%, suggesting that it is unreasonable to use the climate variables of CAS-ESM2-0 to estimate historical PM ETP. Therefore, bias correction must be performed to estimate PM ETP using CAS-ESM2-0.

**Table 3:** Monthly climate variables ranges (Upper, Lower, and Median value) of 14 CMIP6 GCMs in the historical period (1950–2014).

Models	Maximum temperature (°C)			Minimum temperature (°C)			Average temperature (°C)		
	Upper	Lower	Median	Upper	Lower	Median	Upper	Lower	Median
ACCESS-CM2	35.2	−23.4	13.3	20.7	−20.3	7.7	27.7	−24.2	11.5
ACCESS-ESM1-5	34.5	−21.7	14.6	21.1	−16.5	8.7	27.8	−20.7	12.7
CAS-EMS2-0	34.9	−19.5	13.8	20.7	−19.2	7.0	26.7	−20.8	11.6
CMCC-ESM2	33.8	−18.6	13.7	21.5	−17.4	7.4	27.2	−19.2	12.6
CanESM5	36.8	−21.0	13.7	21.0	−19.4	6.9	28.0	−22.2	11.1
FGOALS-g3	35.3	−25.7	13.4	20.1	−22.6	7.3	26.5	−22.8	11.1
GFDL-ESM4	32.1	−19.4	13.0	19.5	−18.1	6.3	25.1	−22.7	11.0
INM-CM4-8	34.5	−22.9	15.4	18.7	−20.0	8.0	25.2	−24.0	12.5
INM-CM5-0	34.7	−21.7	14.8	18.7	−19.0	7.8	25.2	−23.0	12.1
IPSL-CM6A-LR	32.0	−19.5	11.5	19.8	−18.9	6.6	25.5	−21.2	10.1
MIROC6	41.5	−16.0	16.7	22.4	−15.4	9.9	28.6	−17.5	15.2
MPI-ESM1-2-HR	33.9	−20.7	12.4	21.6	−16.9	6.6	26.9	−20.3	12.1
MPI-ESM1-2-LR	33.0	−19.7	12.5	21.3	−17.3	7.4	26.4	−20.2	12.2
MRI-ESM2-0	35.1	−18.0	12.3	21.2	−15.9	7.0	26.7	−18.8	11.6
**Models**	**Solar radiation (W/*m***^2^**)**			**Relative humidity (%)**			**Windspeed (m/s)**		
	**Upper**	**Lower**	**Median**	**Upper**	**Lower**	**Median**	**Upper**	**Lower**	**Median**
ACCESS-CM2	264.1	98.9	216.4	184.3	5.8	112.9	14.5	0.5	8.6
ACCESS-ESM1-5	258.5	98.7	227.1	124.3	10.5	78.8	15.7	0.5	8.6
CAS-EMS2-0	232.8	95.4	196.5	**2628.1**	**11.6**	**130.0**	14.7	0.0	6.8
CMCC-ESM2	250.6	98.1	208.4	154.1	10.6	96.7	16.2	0.6	8.2
CanESM5	261.2	101.8	219.0	99.7	8.6	63.3	15.2	0.6	7.2
FGOALS-g3	259.8	108.2	213.8	105.0	7.0	65.8	14.5	0.6	8.0
GFDL-ESM4	250.7	92.6	208.3	202.7	5.6	123.9	16.2	0.6	7.4
INM-CM4-8	260.9	94.6	228.2	98.4	6.5	61.7	15.4	0.1	7.2
INM-CM5-0	260.2	96.6	225.6	98.7	5.9	61.6	15.4	0.1	7.2
IPSL-CM6A-LR	258.5	102.1	212.0	108.6	9.6	69.0	15.1	0.2	8.1
MIROC6	255.1	100.3	207.4	146.2	7.7	90.8	17.3	0.0	8.1
MPI-ESM1-2-HR	256.6	92.9	218.7	133.2	8.2	83.2	15.4	0.4	7.2
MPI-ESM1-2-LR	254.2	88.6	215.8	132.9	10.5	84.0	16.1	0.5	8.2
MRI-ESM2-0	259.3	103.7	224.4	142.6	4.7	87.4	17.3	0.9	9.6

**Fig. 3:**
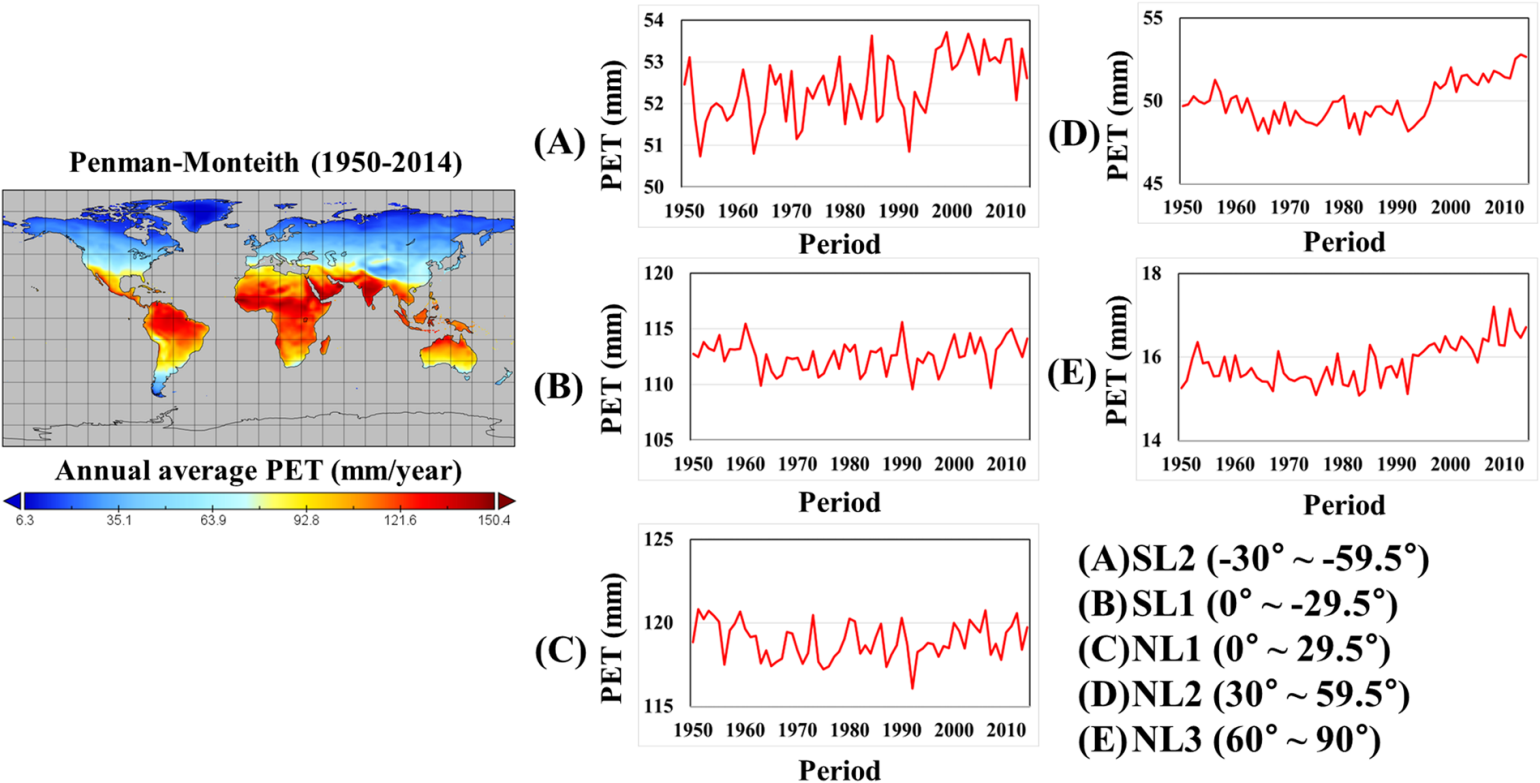
Spatial patterns and temporal changes in annual PM ETP of ACCESS-CM2 in the historical period (1950–2014).

This study developed the Python code to estimate a historical monthly PM ETP sample using climate variables of ACCESS-CM2. Figure 3 presents the spatial and temporal variation of annual PM ETP for ACCESS-CM2 in the historical period. Furthermore, Table 4 presents the historical PM ETP ranges (Upper, Lower, and Median) depending on the 14 CMIP6 GCMs. The PM ETP was generally high in almost areas of NL1 and SL1. and high in some areas of SL2 . In contrast, the PM ETPs at NL2 and NL3 were estimated 3.9-67.0 mm and 2.1-24.2 mm for the historical period, respectively. The PM ETPs at SL2, NL2 and NL3 showed a gradual increase, while there was no significant change at SL1 and NL1. Consequently, the dataset of climate variables generated in this study provides a reasonable estimate of ET over the globe.

**Table 4:** Annual PM ETPs (mm) ranges (Upper, Lower, and Median value) of CMIP6 GCMs in the historical period (1950–2014).

Models	Northern Hemisphere								
	NL1			NL2			NL3		
	Upper	Lower	Median	Upper	Lower	Median	Upper	Lower	Median
ACCESS-CM2	121.4	116.6	119.4	54.4	49.5	51.5	17.4	15.3	16.1
ACCESS-ESM1-5	118.9	114.0	116.5	58.7	53.0	54.9	17.4	15.2	16.1
CAS-EMS2-0	18.9	17.0	17.7	4.6	3.9	4.2	3.4	2.1	2.7
CMCC-ESM2	88.7	83.0	86.5	43.2	37.8	40.3	15.5	10.6	12.7
CanESM5	117.7	111.2	113.7	55.9	50.4	52.8	19.3	17.1	18.1
FGOALS-g3	111.3	106.9	109.1	53.0	48.6	51.0	17.2	14.5	15.7
GFDL-ESM4	107.3	101.2	103.8	49.4	45.5	47.2	16.7	14.7	15.8
INM-CM4-8	119.3	115.1	116.9	64.5	57.5	62.3	20.4	17.5	19.6
INM-CM5-0	118.5	114.4	116.7	62.9	56.0	61.0	21.4	19.0	19.9
IPSL-CM6A-LR	118.9	113.6	116.3	58.2	51.9	54.1	20.0	17.4	18.8
MIROC6	122.8	116.6	120.2	67.0	62.1	64.3	24.2	21.9	22.8
MPI-ESM1-2-HR	122.4	117.5	119.6	57.8	52.0	54.4	17.7	15.5	16.6
MPI-ESM1-2-LR	118.2	113.2	115.8	54.0	48.8	51.3	16.5	14.0	15.0
MRI-ESM2-0	114.4	108.9	110.8	55.6	50.7	52.3	18.7	16.4	17.6
**Models**	**Southern Hemisphere**								
	**SL1**			**SL2**					
	**Upper**	**Lower**	**Median**	**Upper**		**Lower**		**Median**	
ACCESS-CM2	115.0	109.1	112.2	53.0		50.1		51.7	
ACCESS-ESM1-5	121.0	113.0	116.7	54.3		52.3		54.3	
CAS-EMS2-0	13.5	12.0	12.7	1.2		0.4		0.6	
CMCC-ESM2	80.3	74.3	76.8	30.2		26.5		27.9	
CanESM5	115.5	103.4	109.1	49.3		45.9		47.7	
FGOALS-g3	104.7	99.0	102.5	47.7		44.3		46.0	
GFDL-ESM4	101.7	93.6	98.6	46.0		42.0		44.0	
INM-CM4-8	113.2	107.0	110.4	62.3		58.1		60.3	
INM-CM5-0	111.1	106.4	108.5	61.3		56.7		59.3	
IPSL-CM6A-LR	114.7	107.6	111.1	51.2		46.9		48.8	
MIROC6	114.8	105.0	109.6	59.5		55.3		58.0	
MPI-ESM1-2-HR	115.0	109.5	112.0	52.6		47.1		49.1	
MPI-ESM1-2-LR	112.5	105.7	109.4	49.5		46.4		48.2	
MRI-ESM2-0	113.9	109.0	110.7	47.6		44.6		46.5	

For NL1, the historical PM ETP estimated from CanESM5 had the largest difference between the lower and upper bounds, while the PM ETP estimated from INM-CM5-0 had the smallest compared to the other GCMs. The historical PM ETP estimated from INM-CM4-8 had the largest difference between the lower and upper bounds in NL2, and INM-CM5-0 showed the second largest. On the other hand, the historical PM ETP estimated from GFDL-ESM4 had the smallest difference between the lower and upper bounds. For NL3, the difference in PM ETPs estimated from most GCMs between the lower and upper bounds was calculated as 2 to 3, except for CMCC-ESM2. On the other hand, the PM ETP estimated from CMCC-ESM2 was calculated as 4.9 mm, and the difference between the lower and upper bounds was the most significant. For SL1 in SH, the difference in historical PM ETP between the lower and upper bounds estimated from CanESM5 was the largest at 12.1 mm, while PM ETP estimated from INM-CM5-0 had the smallest difference at 4.7 mm. For SL2, the difference in historical PM ETP estimated from MPI-ESM1-2-HR between the lower and upper bounds was the largest at 5.5 mm, while the estimated from ACCESS-ESM1-5 was the smallest. Significantly, the PM ETPs estimated from CAS-ESM2-0 differed from phenomenon in Earth’s latitudes, and PM ETPs of all latitudes needed to be better estimated. Therefore, it is recommended to use a bias correction for estimating PM ETPs using climate variables of CAS-ESM2-0.

### Validation of projected global climate variables and PET for the future period

**Fig. 4:**
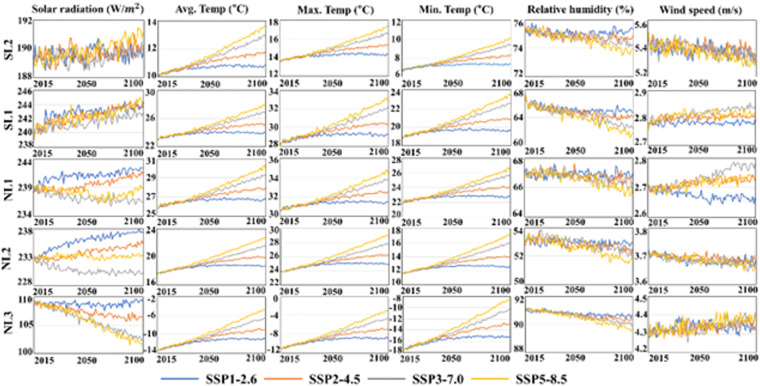
Trends in climate variables of a multi-model ensemble of equally weighted 14 CMIP6 GCMs for the four main SSPs in the future period (2015-2100).

This study compared the projected six climate variables of four SSPs in the future period (2015–2100). Figure 4 presents the climate variables for each SSP scenario generated by equally weighted (0.071) the 14 CMIP6 GCMs. The projected solar radiation increased at most latitudes of SH. In contrast, the projected solar radiation decreased for SSP scenarios with higher greenhouse gas concentrations than those with lower concentrations at most latitudes in NH. The projected trend in wind speed for four SSPs at mid-latitudes (NL2 and SL2) of both hemispheres was lower in the far than in the near futures. However, the projected wind speed at low and high latitudes of both hemispheres was higher for the high-emission scenarios than for the low-emission scenarios. The relative humidity was projected to decrease at all latitudes for all scenarios. Especially the wind speed in NH has decreased the most. Likewise, the decreased signals of relative humidity were lower for the high-emission scenarios than for the low-emission scenarios. All temperatures showed an increase in the future at all latitudes for all SSPs. The increase signals was the highest for SSP5-8.5 at all latitudes compared to other scenarios. The trends in climate variables for different SSPs reflect the greenhouse gas emission levels considered in developing the SSPs.

Supplementary Table S1 presents the upper and lower bounds for future climate variables of SSP scenarios. For relative humidity, most GCMs gradually decrease with higher greenhouse gas concentrations. In particular, the difference in the relative humidity between the upper and lower was the largest in CAS-ESM2-0. Furthermore, the maximum relative humidity of CAS-ESM2-0 was above 200%, and the difference was significant compared to the relative humidity projected by other GCMs. For maximum, minimum, and average temperatures, the MIROC6 was higher than other GCMs. Moreover, the variability of projected future average temperature was the largest in CanESM5, while the variability of ACCESS-CM2 and ACCESS-ESM1-5 was the smallest. Meanwhile, the variability of maximum temperature for all scenarios was the smallest in FGOALS-g3 and MIROC6. On the other hand, the projected minimum temperature for the future was the highest in MIROC6. The variability of minimum temperature was the smallest in ACCESS-ESM1-5, whereas CanESM5 had the highest variability compared to other GCMs. The variability of solar radiation was the smallest for ACCESS-CM2, while MPI-ESM1-2-LR had opposite results. Notably, MPI-ESM1-2-LR had the largest insolation across all scenarios. For wind speed, FGOALS-g3 had the highest variability, while ACCESS-ESM1-5 had the lowest variability.

**Fig. 5:**
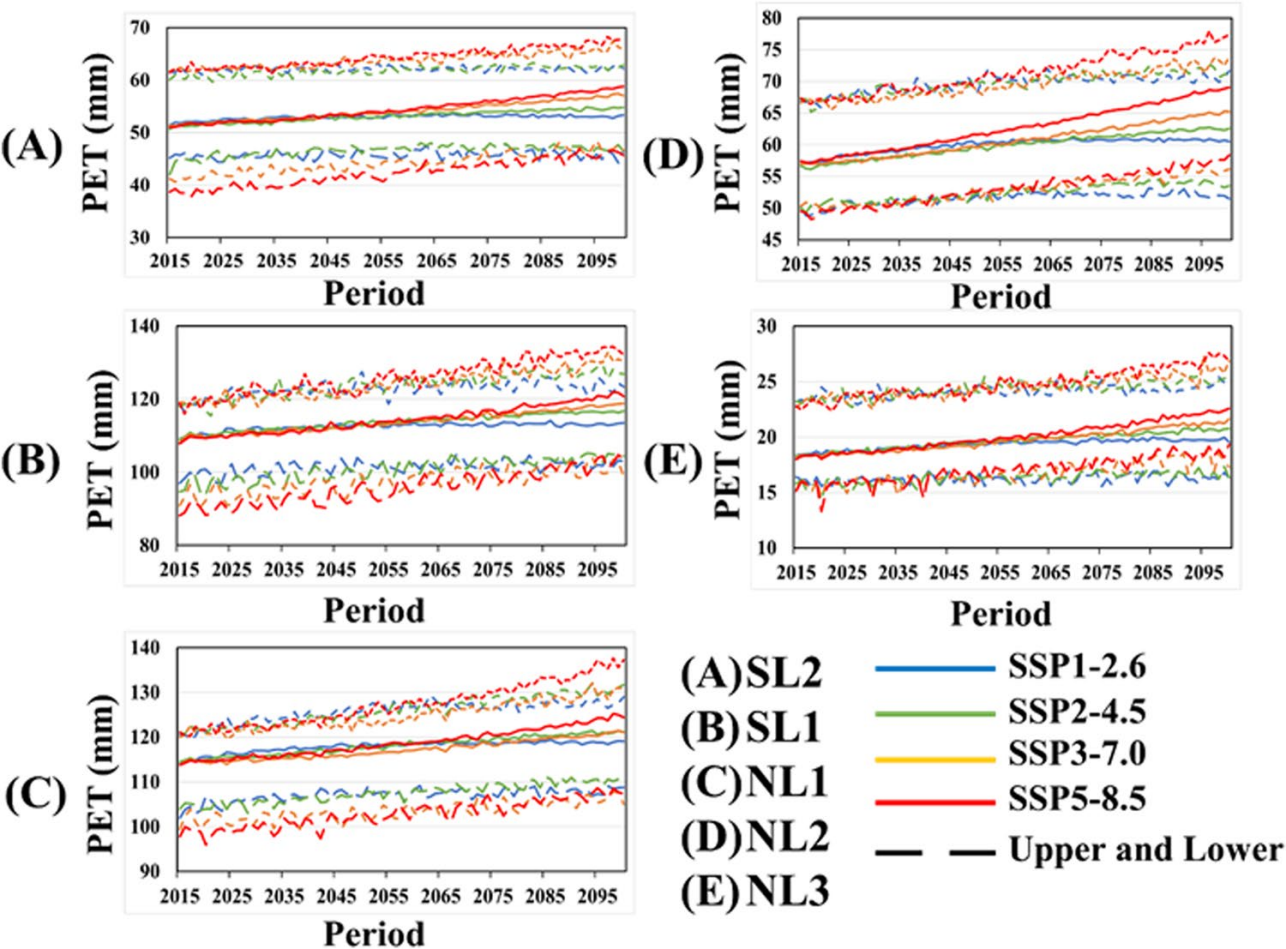
Trends in PM ETPs of 14 CMIP6 GCMs for the four main SSPs in the future period (2015-2100).

This study used the SSP scenarios dataset to project future PM ETP, as shown in Fig. 5. The projected PM ETP showed a gradual increase in all SSPs. The projected PM ETP in high greenhouse gas concentrations increased steeper than in low greenhouse gas concentrations. Furthermore, the upper and lower bounds of projected PM ETP were the highest in SSP5-8.5 in NL2 and NL3, whereas SSP1-2.6 was the lowest. The upper bound of projected PM ETP at all latitudes of SH was the highest in SSP5-8.5. However, the lower bound of projected PM in most scenarios was similar in the far future.

Table 5 presents the projected future annual PM ETP ranges based on the scenarios. Overall, the projected PM ETP of the upper bound in the high greenhouse gas concentration scenario was higher than in the low greenhouse gas scenario. In contrast, the lower bound for PM ETP had the opposite results. For PM ETP estimated from the low-emission scenarios, the difference between the upper and lower bounds was greatest in ACCESS-CM2 compared to the other GCMs, while the difference in GFDL-ESM4 was the smallest. On the other hand, the difference in PET ETP of high emission scenarios between the upper and lower bounds was greatest in ACCESS-CM2 and lowest in CMCC-ESM2. Significantly, the PM ETP estimated from CAS-ESM2-0 was unusually lower compared to the other scenarios, which suggests that the relative humidity projected in CAS-ESM2-0 is unusually high compared to the other GCMs.

**Table 5:** Projected the annual PM ETP (mm) ranges (Upper, Lower, and Median valuse) of main four SSPs in the future period (2015–2100).

Models	SSP1-2.6			SSP2-4.5			SSP3-7.0		
	Upper	Lower	Median	Upper	Lower	Median	Upper	Lower	Median
ACCESS-CM2	129.4	18.6	53.9	131.8	16.6	60.4	132.1	16.8	58.9
ACCESS-ESM1-5	127.5	17.8	57.7	129.6	16.4	63.1	132.8	16.5	62.6
CAS-EMS2-0	6.4	0.0	0.8	14.9	0.2	3.8	20.0	0.6	1.4
CMCC-ESM2	115.0	21.5	54.5	114.1	14.3	59.7	107.8	14.6	55.7
CanESM5	123.1	20.8	55.2	125.6	18.8	61.5	129.1	18.7	62.2
FGOALS-g3	112.4	16.7	52.0	112.4	15.6	53.7	115.2	16.0	55.5
GFDL-ESM4	109.2	17.0	48.8	111.9	19.4	52.5	111.4	16.3	52.9
INM-CM4-8	122.4	20.5	63.3	132.1	16.8	62.6	126.4	19.6	67.4
INM-CM5-0	121.4	21.3	62.4	123.2	20.0	65.6	125.8	20.0	66.2
IPSL-CM6A-LR	121.6	20.8	56.8	123.9	19.1	61.4	125.2	19.1	61.5
MIROC6	126.9	24.2	65.9	127.6	22.7	69.9	126.8	22.6	69.4
MPI-ESM1-2-HR	124.2	18.0	55.9	125.0	16.6	59.0	126.1	16.8	59.4
MPI-ESM1-2-LR	120.2	16.3	51.7	121.0	15.2	56.2	121.8	14.7	56.5
MRI-ESM2-0	119.1	19.2	55.6	120.8	18.1	59.0	122.0	17.7	58.2
**Models**	**SSP5-8.5**								
	**Upper**			**Lower**			**Median**		
ACCESS-CM2	137.6			16.5			61.2		
ACCESS-ESM1-5	134.3			16.9			64.4		
CAS-EMS2-0	32.2			1.5			10.1		
CMCC-ESM2	108.6			13.3			55.2		
CanESM5	134.2			18.8			64.1		
FGOALS-g3	115.2			16.0			55.5		
GFDL-ESM4	113.4			16.1			53.2		
INM-CM4-8	129.7			19.6			68.8		
INM-CM5-0	129.1			20.3			67.6		
IPSL-CM6A-LR	129.2			19.3			63.3		
MIROC6	131.0			22.4			71.2		
MPI-ESM1-2-HR	127.3			16.7			60.1		
MPI-ESM1-2-LR	123.7			15.2			57.1		
MRI-ESM2-0	124.9			18.0			60.2		

### Projected changes in annual and seasonal potential evapotranspiration

**Fig. 6:**
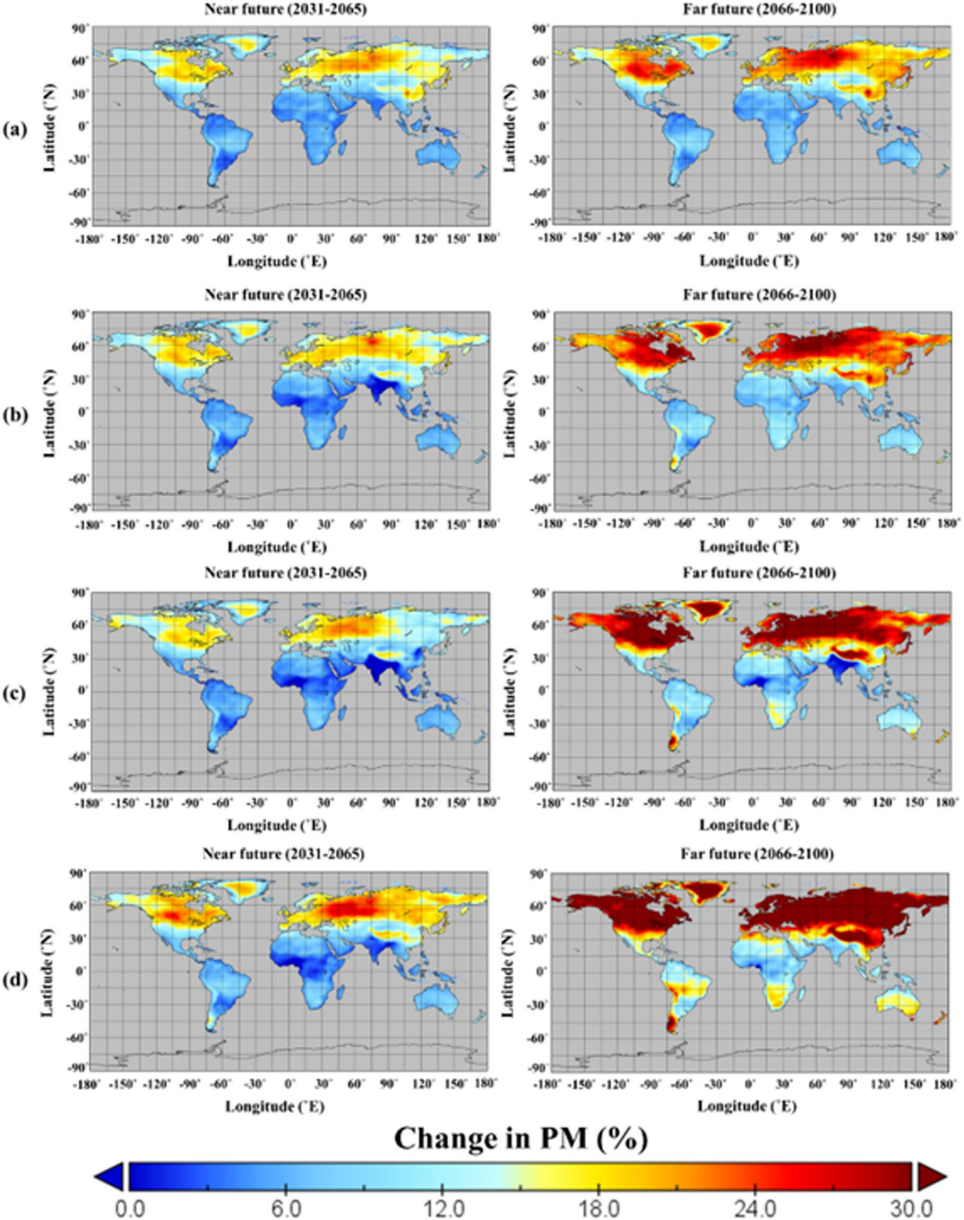
Spatial and temporal changes (%) in projected annual PM ETP estimated for four SSPs based on the equal-weighted multi-model ensemble compared to the base period (1980–2014) (**a**: SSP1-2.6, **b**:SSP2-4.5, **c**: SSP3-7.0, and **d**: SSP5-8.5).

The spatially interpolated changes in annual PM ETP for different SSP scenarios are shown in Fig. 6. Overall, PM ETP for all scenarios at NL2 showed an increase in the near future compared to the historical period. Furthermore, the increased signals were more in the far future than in the near future. Especially the change in PM ETP for the high-emission scenarios at all latitudes was higher than for the low-emission scenarios. Therefore, the PM ETP changes at all latitudes were aligned with the emission levels of SSPs. Supplementary Table S2 shows the range of change in annual and seasonal PM ETP based on the four SSP scenarios. For SSP1-2.6, the annual and seasonal PM ETP of CMCC-ESM2-0 had the largest variability, while the PM ETP of CAS-ESM2-0 had decreased compared to the historical period. Furthermore, the annual PM ETP of INM-CM4-8 had the smallest variability. Furthermore, the variability of seasonal PM ETP was the smallest in INM-CM4-8 (Winter), FGOALS-g3 (Spring), and MPI-ESM1-2-LR (Fall), respectively. For SSP2-4.5, the variability of annual and seasonal PM ETP was the smallest for FGOALS-g3, while CMCC-ESM2-0 was the opposite results. Meanwhile, the variability of annual and seasonal PM ETP estimated in SSP3-7.0 was the smallest for INM-CM4-8, and the variability of annual PM ETP for SSP5-8.5 was also the lowest for INM-CM4-8. On the other hand, the GCMs with lower seasonal variability for SSP5-8.5 were all estimated differently.

## Usage Notes

This global dataset can improve robust projections of the future climate for SSPs using various GCMs. It can be used to analyze the climate change impact and quantify the effectiveness of adaptation and mitigation policies. Its applicability can be extended in the future by adding simulations for more GCMs and SSPs.

### Supplementary information

Supplementary Information

## Data Availability

The code to produce the data was written using Python, PyCharm 2022.2.2. The code is available in pyeto^30^ (https://pyeto.readthedocs.io/en/latest/).
