# Supplementary material for: Development of global monthly dataset of CMIP6 climate variables for estimating evapotranspiration
Source: Sci Data. 2023 Aug 26;10:568. doi: 10.1038/s41597-023-02475-7 (PMC10460419; doi:10.1038/s41597-023-02475-7)
Supplement: Supplementary file 1 — Supplementary Information [file 41597_2023_2475_MOESM1_ESM.docx]

**Supplementary Information**

**Development of global monthly dataset of CMIP6 climate variables for estimating evapotranspiration**

### Authors

Young Hoon Song^1^, Eun-Sung Chung^2^ , Shamsuddin Shahid^3^ , Yeonjoo Kim^4^ , Dongkyun Kim^5^ ^†^

**Affiliations**

1. Department of Civil Engineering, Seoul National University of Science and Technology, Nowon-gu, 01811, Seoul, South Korea

2. Department of Civil Engineering, Seoul National University of Science and Technology, Nowon-gu, 01811, Seoul, South Korea. Correspondence and requests for materials should be addressed to E.-S.C. (email: eschung@seoultech.ac.kr)

3. School of Civil Engineering, Universiti Teknologi Malaysia (UTM), 81310, Skudai, Johor, Malaysia

4. Department of Civil and Environmental Engineering, Yonsei University, 03722, Seoul, South Korea

5. Department of Civil Engineering, Hongik University, 04066, Seoul, South Korea

**Supplementary Table S1**. Projected the monthly climate variables ranges (Upper and Lower) of main four SSPs by latitude band in the future period (2015-2100)

| Models | Relative humidity (%) | | | | | | | |
| --- | --- | --- | --- | --- | --- | --- | --- | --- |
|  | SSP1-2.6 | | SSP2-4.5 | | SSP3-7.0 | | SSP5-8.5 | |
|  | Lower | Upper | Lower | Upper | Lower | Upper | Lower | Upper |
| ACCESS-CM2 | 3.5 | 191.6 | 3.9 | 189.4 | 3.7 | 192.0 | 3.5 | 189.1 |
| ACCESS-ESM1-5 | 3.1 | 172.4 | 3.4 | 170.5 | 3.9 | 169.9 | 2.6 | 168.2 |
| CanESM5 | 2.2 | 115.6 | -0.7 | 115.3 | -0.4 | 114.9 | 0.2 | 116.8 |
| **CAS-ESM2-0** | **-137.6** | **7002.5** | **-70.4** | **4506.2** | **-81.9** | **5150.5** | **4.2** | **5679.7** |
| CMCC-ESM2-0 | -5.5 | 430.9 | -5.2 | 524.0 | -4.8 | 648.3 | -5.4 | 772.9 |
| FGOALS-g3 | 5.5 | 107.5 | 5.4 | 106.2 | 3.7 | 107.8 | 5.4 | 107.0 |
| GFDL-ESM4 | 2.3 | 390.9 | 2.3 | 347.0 | 2.4 | 327.0 | 2.2 | 356.8 |
| INM-CM4-8 | 1.4 | 104.2 | 1.4 | 101.9 | 0.8 | 103.2 | 1.4 | 101.3 |
| INM-CM5-0 | 1.5 | 101.3 | 1.7 | 101.0 | 1.6 | 100.5 | 1.1 | 102.6 |
| IPSL-CM6A-LR | 2.8 | 109.6 | 3.7 | 111.0 | 4.9 | 107.7 | 3.5 | 108.3 |
| MIROC6 | 2.2 | 203.0 | 2.2 | 211.3 | 2.1 | 207.4 | 2.2 | 211.4 |
| MPI-ESM1-2-HR | 5.1 | 143.6 | 4.4 | 144.2 | 5.0 | 143.6 | 5.0 | 144.6 |
| MPI-ESM1-2-LR | 4.7 | 151.0 | 0.9 | 152.8 | 0.3 | 154.0 | 4.7 | 153.7 |
| MRI-ESM2-0 | 3.4 | 162.0 | 3.7 | 157.0 | 3.6 | 159.8 | 3.4 | 160.5 |
| Models | Average temperature (℃) | | | | | | | |
|  | SSP1-2.6 | | SSP2-4.5 | | SSP3-7.0 | | SSP5-8.5 | |
|  | Lower | Upper | Lower | Upper | Lower | Upper | Lower | Upper |
| ACCESS-CM2 | -81.4 | 44.9 | -80.4 | 46.0 | -76.4 | 46.8 | -78.8 | 48.9 |
| ACCESS-ESM1-5 | -68.6 | 42.6 | -66.5 | 43.5 | -66.8 | 45.8 | -66.2 | 46.3 |
| CanESM5 | -84.9 | 44.4 | -82.8 | 46.2 | -84.3 | 48.4 | -83.6 | 49.8 |
| CAS-ESM2-0 | -76.2 | 47.1 | -77.2 | 48.5 | -77.5 | 49.4 | -76.4 | 51.5 |
| CMCC-ESM2-0 | -82.8 | 47.5 | -80.1 | 48.8 | -80.4 | 49.9 | -79.2 | 52.0 |
| FGOALS-g3 | -69.1 | 42.8 | -68.0 | 43.8 | -68.0 | 45.2 | -68.6 | 45.5 |
| GFDL-ESM4 | -73.8 | 45.4 | -72.8 | 46.5 | -72.6 | 48.7 | -73.3 | 49.5 |
| INM-CM4-8 | -78.7 | 42.6 | -77.3 | 43.8 | -77.8 | 45.6 | -79.9 | 46.5 |
| INM-CM5-0 | -78.8 | 41.6 | -78.3 | 42.7 | -79.1 | 44.0 | -78.6 | 45.2 |
| IPSL-CM6A-LR | -81.7 | 42.4 | -79.0 | 44.5 | -79.9 | 47.0 | -80.3 | 49.0 |
| MIROC6 | **-70.6** | **55.2** | **-70.6** | **56.2** | **-71.4** | **57.4** | **-72.1** | **58.8** |
| MPI-ESM1-2-HR | -73.7 | 46.1 | -73.0 | 48.2 | -72.4 | 49.2 | -72.8 | 49.9 |
| MPI-ESM1-2-LR | -76.7 | 45.7 | -80.8 | 46.8 | -77.2 | 49.1 | -78.9 | 50.2 |
| MRI-ESM2-0 | -65.8 | 47.2 | -65.1 | 47.5 | -64.5 | 48.8 | -65.2 | 50.6 |
| Models | Maximum temperature (℃) | | | | | | | |
|  | SSP1-2.6 | | SSP2-4.5 | | SSP3-7.0 | | SSP5-8.5 | |
|  | Lower | Upper | Lower | Upper | Lower | Upper | Lower | Upper |
| ACCESS-CM2 | -79.6 | 51.2 | -77.0 | 52.8 | -74.3 | 54.2 | -77.2 | 56.2 |
| ACCESS-ESM1-5 | -65.3 | 54.7 | -63.7 | 55.1 | -63.3 | 56.9 | -63.0 | 57.7 |
| CanESM5 | -80.0 | 54.9 | -78.7 | 56.9 | -81.3 | 60.0 | -80.0 | 61.2 |
| CAS-ESM2-0 | -74.1 | 53.9 | -74.3 | 54.8 | -74.4 | 56.0 | -74.0 | 58.3 |
| CMCC-ESM2-0 | -77.9 | 52.6 | -74.3 | 53.7 | -74.9 | 55.1 | -75.0 | 56.9 |
| FGOALS-g3 | -64.5 | 50.7 | -64.4 | 51.5 | -64.0 | 52.1 | -64.4 | 52.7 |
| GFDL-ESM4 | -71.4 | 51.5 | -71.0 | 52.8 | -70.2 | 54.8 | -71.1 | 54.9 |
| INM-CM4-8 | -76.7 | 53.7 | -75.6 | 53.2 | -76.2 | 55.5 | -78.2 | 55.8 |
| INM-CM5-0 | -76.8 | 52.9 | -76.7 | 52.4 | -77.3 | 53.7 | -77.7 | 54.5 |
| IPSL-CM6A-LR | -77.9 | 56.6 | -75.0 | 53.1 | -75.7 | 56.0 | -75.9 | 61.3 |
| MIROC6 | **-68.1** | **72.6** | **-68.5** | **73.0** | **-69.5** | **74.0** | **-69.3** | **76.1** |
| MPI-ESM1-2-HR | -72.4 | 53.1 | -71.6 | 54.9 | -71.5 | 55.9 | -72.7 | 56.4 |
| MPI-ESM1-2-LR | -76.2 | 50.7 | -80.6 | 52.1 | -76.8 | 54.9 | -78.0 | 55.9 |
| MRI-ESM2-0 | -64.7 | 55.5 | -63.9 | 55.5 | -63.3 | 57.1 | -64.4 | 58.6 |
| Models | Minimum temperature (℃) | | | | | | | |
|  | SSP1-2.6 | | SSP2-4.5 | | SSP3-7.0 | | SSP5-8.5 | |
|  | Lower | Upper | Lower | Upper | Lower | Upper | Lower | Upper |
| ACCESS-CM2 | -83.3 | 38.6 | -83.4 | 40.7 | -79.6 | 41.5 | -80.7 | 43.7 |
| ACCESS-ESM1-5 | -71.7 | 36.4 | -69.4 | 37.2 | -70.3 | 38.6 | -71.5 | 39.7 |
| CanESM5 | -90.3 | 38.3 | -87.2 | 40.6 | -87.7 | 42.3 | -88.5 | 44.2 |
| CAS-ESM2-0 | -78.6 | 41.7 | -80.0 | 42.9 | -81.2 | 44.2 | -78.9 | 46.0 |
| CMCC-ESM2-0 | -88.3 | 42.4 | -85.9 | 43.8 | -85.4 | 44.6 | -84.7 | 47.2 |
| FGOALS-g3 | -76.3 | 36.9 | -75.9 | 38.1 | -74.9 | 39.6 | -76.3 | 40.3 |
| GFDL-ESM4 | -76.4 | 38.2 | -75.1 | 39.5 | -74.8 | 41.5 | -75.5 | 42.6 |
| INM-CM4-8 | -80.6 | 39.0 | -79.5 | 39.2 | -79.3 | 39.4 | -82.3 | 39.5 |
| INM-CM5-0 | -80.9 | 38.4 | -80.0 | 38.9 | -81.0 | 39.3 | -80.1 | 39.4 |
| IPSL-CM6A-LR | -84.6 | 36.1 | -83.0 | 37.5 | -83.7 | 39.8 | -84.3 | 42.9 |
| MIROC6 | **-73.2** | **43.9** | **-73.0** | **44.5** | **-73.2** | **46.6** | **-74.9** | **48.4** |
| MPI-ESM1-2-HR | -75.0 | 40.0 | -74.5 | 41.8 | -73.7 | 43.0 | -74.0 | 43.7 |
| MPI-ESM1-2-LR | -77.1 | 40.8 | -81.0 | 41.6 | -77.4 | 43.1 | -79.2 | 44.5 |
| MRI-ESM2-0 | -67.2 | 42.4 | -66.7 | 43.3 | -65.7 | 44.4 | -66.0 | 45.8 |
| Models | Solar radiation $(W/m^{2})$ | | | | | | | |
|  | SSP1-2.6 | | SSP2-4.5 | | SSP3-7.0 | | SSP5-8.5 | |
|  | Lower | Upper | Lower | Upper | Lower | Upper | Lower | Upper |
| ACCESS-CM2 | 0 | 462.3 | 0 | 462.8 | 0 | 459.3 | 0 | 461.5 |
| ACCESS-ESM1-5 | 0 | 478.9 | 0 | 474.8 | 0 | 476.3 | 0 | 476.4 |
| CanESM5 | 0 | 506.8 | 0 | 509.6 | 0 | 504.3 | 0 | 491.9 |
| CAS-ESM2-0 | 0 | 466.3 | 0 | 466.4 | 0 | 466.2 | 0 | 468.4 |
| CMCC-ESM2-0 | 0 | 468.1 | 0 | 470.8 | 0 | 468.2 | 0 | 469.2 |
| FGOALS-g3 | 0 | 466.5 | 0 | 463.9 | 0 | 464.9 | 0 | 466.8 |
| GFDL-ESM4 | 0 | 479.2 | 0 | 481.6 | 0 | 479.4 | 0 | 480.6 |
| INM-CM4-8 | 0 | 468.8 | 0 | 466.2 | 0 | 462.2 | 0 | 463.0 |
| INM-CM5-0 | 0 | 463.9 | 0 | 464.7 | 0 | 466.4 | 0 | 465.5 |
| IPSL-CM6A-LR | 0 | 478.8 | 0 | 480.7 | 0 | 481.2 | 0 | 477.4 |
| MIROC6 | 0 | 468.1 | 0 | 471.4 | 0 | 471.3 | 0 | 465.2 |
| MPI-ESM1-2-HR | 0 | 533.5 | 0 | 535.2 | 0 | 541.0 | 0 | 533.3 |
| MPI-ESM1-2-LR | 0 | 565.2 | 0 | 558.6 | 0 | 557.0 | 0 | 558.8 |
| MRI-ESM2-0 | 0 | 476.0 | 0 | 484.6 | 0 | 475.6 | 0 | 477.7 |
| Models | Wind speed ($m/s$) | | | | | | | |
|  | SSP1-2.6 | | SSP2-4.5 | | SSP3-7.0 | | SSP5-8.5 | |
|  | Lower | Upper | Lower | Upper | Lower | Upper | Lower | Upper |
| ACCESS-CM2 | 0.4 | 18.9 | 0.4 | 19.7 | 0.4 | 18.4 | 0.4 | 19.6 |
| ACCESS-ESM1-5 | 0.4 | 28.8 | 0.4 | 28.2 | 0.4 | 29.0 | 0.4 | 28.6 |
| CanESM5 | 0.5 | 17.8 | 0.5 | 17.3 | 0.5 | 18.1 | 0.5 | 17.4 |
| CAS-ESM2-0 | 0.0 | 16.8 | 0.0 | 16.7 | 0.0 | 16.9 | 0.0 | 17.5 |
| CMCC-ESM2-0 | 0.6 | 18.3 | 0.6 | 18.0 | 0.6 | 18.1 | 0.5 | 18.7 |
| FGOALS-g3 | 0.4 | 17.7 | 0.4 | 16.9 | 0.4 | 16.5 | 0.4 | 17.7 |
| GFDL-ESM4 | 0.4 | 19.1 | 0.4 | 18.7 | 0.4 | 19.4 | 0.4 | 19.2 |
| INM-CM4-8 | 0.0 | 17.8 | 0.0 | 17.4 | 0.0 | 17.5 | 0.0 | 17.3 |
| INM-CM5-0 | 0.0 | 17.8 | 0.0 | 17.2 | 0.0 | 17.3 | 0.0 | 17.3 |
| IPSL-CM6A-LR | 0.0 | 16.8 | 0.0 | 16.8 | 0.0 | 16.8 | 0.0 | 16.4 |
| MIROC6 | 0.0 | 18.4 | 0.0 | 18.7 | 0.0 | 18.4 | 0.0 | 19.6 |
| MPI-ESM1-2-HR | 0.3 | 17.8 | 0.3 | 18.6 | 0.3 | 19.2 | 0.3 | 18.3 |
| MPI-ESM1-2-LR | 0.0 | 17.9 | 0.0 | 17.6 | 0.0 | 17.5 | 0.0 | 17.3 |
| MRI-ESM2-0 | 0.8 | 27.7 | 0.8 | 27.2 | 0.8 | 27.3 | 0.8 | 27.3 |

**Supplementary Table S2.** Changes (%) in the future annual and seasonality PM ETP ranges (Upper and Lower) of the main four SSPs compared to the base period (1980-2014)

| Models | SSP1-2.6 | | | | | | | | | |
| --- | --- | --- | --- | --- | --- | --- | --- | --- | --- | --- |
|  | Annual | | Winter | | Spring | | Summer | | Fall | |
|  | Upper | Lower | Upper | Lower | Upper | Lower | Upper | Lower | Upper | Lower |
| ACCESS-CM2 | 17.7 | 5.6 | 36.3 | 4.6 | 15.4 | 5.6 | 17.0 | 5.0 | 24.5 | 5.9 |
| ACCESS-ESM1-5 | 13.6 | 3.8 | 41.8 | 3.9 | 14.5 | 2.7 | 11.9 | 4.1 | 16.0 | 3.7 |
| CanESM5 | 15.0 | 5.2 | 104.1 | 5.3 | 23.3 | 4.5 | 13.6 | 4.2 | 24.5 | 4.6 |
| CAS-ESM2-0 | -74.4 | -99.2 | -83.5 | -100.0 | -82.3 | -99.9 | -72.2 | -100.0 | -80.5 | -100.0 |
| CMCC-ESM2-0 | 200+ | 31.5 | 200+ | 31.5 | 200+ | 29.4 | 200+ | 34.5 | 200+ | 30.1 |
| FGOALS-g3 | 6.1 | 1.4 | 11.5 | 1.9 | 8.6 | 1.3 | 5.4 | 1.6 | 7.9 | 0.8 |
| GFDL-ESM4 | 12.1 | 3.8 | 36.1 | 3.3 | 14.1 | 3.8 | 9.9 | 3.6 | 16.2 | 4.1 |
| INM-CM4-8 | 6.4 | 1.8 | 12.1 | 1.6 | 8.2 | 2.0 | 5.7 | 1.6 | 9.8 | 1.9 |
| INM-CM5-0 | 7.5 | 2.0 | 10.6 | 1.4 | 9.3 | 2.3 | 6.6 | 1.8 | 21.5 | 2.2 |
| IPSL-CM6A-LR | 11.5 | 2.6 | 54.9 | 1.9 | 12.7 | 2.7 | 10.2 | 2.2 | 19.1 | 2.1 |
| MIROC6 | 9.8 | 2.5 | 46.7 | 2.4 | 13.9 | 2.3 | 8.3 | 1.6 | 10.4 | 2.2 |
| MPI-ESM1-2-HR | 25.2 | 18.6 | 66.3 | 17.9 | 30.8 | 18.9 | 24.0 | 17.7 | 25.6 | 18.2 |
| MPI-ESM1-2-LR | 7.9 | 1.8 | 46.2 | 1.3 | 13.0 | 2.4 | 7.5 | 0.7 | 7.0 | 1.2 |
| MRI-ESM2-0 | 11.7 | 4.2 | 104.2 | 4.0 | 12.7 | 3.7 | 10.5 | 3.4 | 17.1 | 2.8 |
| Models | SSP2-4.5 | | | | | | | | | |
|  | Annual | | Winter | | Spring | | Summer | | Fall | |
|  | Upper | Lower | Upper | Lower | Upper | Lower | Upper | Lower | Upper | Lower |
| ACCESS-CM2 | 20.9 | 4.6 | 41.5 | 5.9 | 20.2 | 4.7 | 20.6 | 3.9 | 34.0 | 4.5 |
| ACCESS-ESM1-5 | 17.0 | 4.5 | 72.8 | 4.2 | 17.7 | 4.5 | 15.3 | 3.7 | 20.3 | 4.9 |
| CanESM5 | 20.8 | 4.0 | 209.9 | 5.6 | 28.9 | 4.1 | 18.0 | 2.6 | 33.5 | 3.7 |
| CAS-ESM2-0 | 200+ | 32.6 | 200+ | 55.9 | 144.8 | 52.5 | 24.1 | 21.7 | 200+ | 72.2 |
| CMCC-ESM2-0 | 200+ | 26.8 | 200+ | 32.6 | 200+ | 24.9 | 200+ | 28.5 | 200+ | 25.5 |
| FGOALS-g3 | 6.1 | 1.4 | 11.5 | 1.9 | 8.6 | 1.3 | 5.4 | 1.6 | 7.9 | 0.8 |
| GFDL-ESM4 | 16.2 | 3.7 | 76.3 | 4.4 | 17.4 | 3.8 | 28.2 | 2.6 | 27.2 | 3.5 |
| INM-CM4-8 | 8.9 | 3.1 | 17.8 | 3.2 | 11.1 | 3.1 | 8.2 | 2.7 | 13.7 | 2.8 |
| INM-CM5-0 | 9.9 | 3.0 | 36.8 | 3.0 | 13.9 | 2.7 | 8.8 | 2.4 | 15.3 | 2.4 |
| IPSL-CM6A-LR | 16.9 | 2.3 | 81.7 | 1.8 | 19.5 | 2.9 | 15.3 | 1.5 | 29.7 | 1.7 |
| MIROC6 | 11.1 | 1.7 | 39.3 | 2.2 | 18.7 | 2.1 | 9.5 | 1.0 | 11.9 | 1.3 |
| MPI-ESM1-2-HR | 28.7 | 17.9 | 77.0 | 18.4 | 36.5 | 18.8 | 27.4 | 17.3 | 29.8 | 17.6 |
| MPI-ESM1-2-LR | 12.2 | 1.3 | 59.4 | 1.9 | 18.8 | 2.8 | 11.4 | 0.2 | 11.2 | 0.4 |
| MRI-ESM2-0 | 14.3 | 3.6 | 22.6 | 3.7 | 16.3 | 3.7 | 13.0 | 2.5 | 188.3 | 3.4 |
| Models | SSP3-7.0 | | | | | | | | | |
|  | Annual | | Winter | | Spring | | Summer | | Fall | |
|  | Upper | Lower | Upper | Lower | Upper | Lower | Upper | Lower | Upper | Lower |
| ACCESS-CM2 | 24.6 | 3.4 | 60.9 | 5.1 | 23.8 | 3.9 | 24.2 | 2.6 | 38.8 | 2.6 |
| ACCESS-ESM1-5 | 19.6 | 3.6 | 90.8 | 4.2 | 23.1 | 3.3 | 18.0 | 3.2 | 24.3 | 3.9 |
| CanESM5 | 26.2 | 3.1 | 439.2 | 6.3 | 36.1 | 4.0 | 22.8 | 1.0 | 44.5 | 2.3 |
| CAS-ESM2-0 | 200+ | -15.7 | 63.8 | -100.0 | 77.4 | 5.3 | 200+ | -14.9 | 200+ | -100.0 |
| CMCC-ESM2-0 | 200+ | 20.5 | 200+ | 23.5 | 200+ | 19.5 | 200+ | 21.6 | 200+ | 18.6 |
| FGOALS-g3 | 16.9 | 1.4 | 89.0 | 2.4 | 28.0 | 1.7 | 13.6 | 0.8 | 25.9 | 1.3 |
| GFDL-ESM4 | 18.4 | 2.9 | 103.5 | 5.2 | 21.4 | 3.4 | 40.2 | 1.3 | 37.4 | 2.4 |
| INM-CM4-8 | 11.5 | 3.0 | 36.2 | 3.0 | 14.7 | 3.0 | 10.6 | 2.9 | 19.3 | 2.9 |
| INM-CM5-0 | 12.6 | 3.2 | 53.5 | 3.6 | 17.8 | 3.3 | 11.4 | 2.6 | 19.8 | 2.8 |
| IPSL-CM6A-LR | 22.4 | 1.9 | 128.7 | 0.9 | 25.8 | 3.3 | 20.3 | 1.3 | 40.4 | 1.1 |
| MIROC6 | 12.2 | 1.2 | 59.0 | 2.0 | 23.1 | 1.5 | 10.1 | 0.6 | 15.2 | 0.7 |
| MPI-ESM1-2-HR | 34.0 | 17.3 | 105.0 | 17.9 | 45.5 | 18.3 | 31.5 | 16.5 | 34.7 | 16.9 |
| MPI-ESM1-2-LR | 16.5 | 0.6 | 68.7 | 2.0 | 25.9 | 1.9 | 16.0 | -0.8 | 15.6 | -0.2 |
| MRI-ESM2-0 | 14.7 | 2.1 | 206.9 | 2.6 | 21.6 | 2.7 | 13.3 | 0.7 | 25.1 | 1.6 |
| Models | SSP5-8.5 | | | | | | | | | |
|  | Annual | | Winter | | Spring | | Summer | | Fall | |
|  | Upper | Lower | Upper | Lower | Upper | Lower | Upper | Lower | Upper | Lower |
| ACCESS-CM2 | 31.3 | 4.9 | 89.0 | 5.5 | 32.0 | 5.1 | 30.1 | 4.5 | 49.8 | 5.1 |
| ACCESS-ESM1-5 | 24.8 | 5.3 | 131.1 | 5.0 | 29.5 | 5.2 | 22.3 | 4.2 | 30.7 | 6.0 |
| CanESM5 | 32.9 | 5.1 | 581.7 | 6.7 | 42.2 | 5.5 | 28.3 | 3.2 | 52.6 | 4.8 |
| CAS-ESM2-0 | 200+ | -56.8 | 0.2 | -99.9 | -12.8 | -65.7 | -27.1 | -100.0 | -29.1 | -100.0 |
| CMCC-ESM2-0 | 200+ | 19.3 | 200+ | 20.4 | 200+ | 18.3 | 200+ | 20.5 | 200+ | 17.8 |
| FGOALS-g3 | 16.9 | 1.4 | 89.0 | 2.4 | 28.0 | 1.7 | 13.6 | 0.8 | 25.9 | 1.3 |
| GFDL-ESM4 | 20.4 | 3.3 | 84.1 | 4.3 | 24.1 | 3.2 | 26.1 | 2.2 | 29.4 | 3.4 |
| INM-CM4-8 | 15.5 | 4.4 | 61.1 | 4.5 | 20.3 | 4.4 | 14.3 | 3.6 | 25.6 | 4.0 |
| INM-CM5-0 | 15.9 | 4.3 | 60.8 | 4.6 | 21.6 | 4.3 | 14.5 | 3.6 | 24.6 | 2.9 |
| IPSL-CM6A-LR | 30.0 | 2.4 | 191.3 | 1.6 | 33.6 | 3.4 | 27.6 | 1.9 | 52.8 | 1.2 |
| MIROC6 | 16.8 | 2.4 | 72.0 | 3.0 | 27.8 | 2.5 | 13.8 | 1.1 | 18.5 | 2.1 |
| MPI-ESM1-2-HR | 37.3 | 17.3 | 108.3 | 18.2 | 50.9 | 19.1 | 35.1 | 15.6 | 40.3 | 16.7 |
| MPI-ESM1-2-LR | 20.2 | 0.7 | 75.7 | 2.1 | 30.7 | 2.7 | 19.6 | -1.0 | 20.6 | -0.4 |
| MRI-ESM2-0 | 19.8 | 4.6 | 200+ | 4.5 | 24.4 | 4.6 | 17.9 | 2.8 | 29.9 | 4.7 |
